# Genome-wide homozygosity signature and risk of Hodgkin lymphoma

**DOI:** 10.1038/srep14315

**Published:** 2015-09-22

**Authors:** Amit Sud, Rosie Cooke, Anthony J. Swerdlow, Richard S. Houlston

**Affiliations:** 1 Division of Genetics and Epidemiology, The Institute of Cancer Research, London, UK; 2 Division of Breast Cancer Research, The Institute of Cancer Research, London, UK

## Abstract

Recent studies have reported that regions of homozygosity (ROH) in the genome are detectable in outbred populations and can be associated with an increased risk of malignancy. To examine whether homozygosity is associated with an increased risk of developing Hodgkin lymphoma (HL) we analysed 589 HL cases and 5,199 controls genotyped for 484,072 tag single nucleotide polymorphisms (SNPs). Across the genome the cumulative distribution of ROH was not significantly different between cases and controls. Seven ROH at 4q22.3, 4q32.2, 7p12.3–14.1, 7p22.2, 10p11.22–23, 19q13.12-2 and 19p13.2 were associated with HL risk at *P* < 0.01. Intriguingly 4q22.3 harbours an ROH to which the nuclear factor NF-kappa-B p105 subunit (*NFKB1*) maps (*P* = 0.002). The ROH at 19q13.12-2 has previously been implicated in B-cell precursor acute lymphoblastic leukaemia. Aside from these observations which require validation, it is unlikely that levels of measured homozygosity caused by autozygosity, uniparental isodisomy or hemizygosity play a major role in defining HL risk in predominantly outbred populations.

Hodgkin lymphoma (HL) is a B-cell malignancy which affects around 3 per 100,000 of the population each year in most western countries^1^. The cancer is conventionally classified based on its histopathogy into classical HL (cHL), which accounts for 95% of cases, and nodular lymphocyte predominant HL (NLPHL). The presence of Epstein-Barr virus (EBV) latent membrane protein and/or EBV-encoded small RNAs (EBERs) in Hodgkin Reed-Sternberg cells (HRS) defines EBV-positive disease^2^.

Evidence for inherited genetic predisposition to HL is provided by the three to nine-fold increased risk of HL in first-degree relatives of HL patients^3^. To date, no high penetrance susceptibility loci have been identified for HL and most of the inherited risk is likely to be the consequence of the co-inheritance of multiple low-risk variants. Genome-wide association studies (GWAS) of HL have identified several common (minor allele frequency [MAF] >5%) single nucleotide polymorphisms (SNPs), at 2p16.1 (*REL*), 3p24.1 (*EOMES*), 6q23.3 (*HBSL1L, MYB*), 8q24.21 (PVT1), 10p14 (GATA3), 19p13.3 (*TCF3*), as well as multiple associations within 6p21 (HLA region)^4^,^5^,^6^,^7^,^8^,^9^. While SNPs (excluding some located in the HLA region) individually confer small effects (odds ratio [OR] <1.6) on HL risk they have given novel insights into HL biology implicating perturbation of the Rel/NF-κB pathways in disease aetiology.

The majority of cancer predisposition SNPs that have been identified to date through GWAS act in a co-dominant fashion and studies have found no good evidence for recessively acting alleles^10^. Although this may be reflective of the biology, it may also be a consequence of GWAS having suboptimal ability to detect recessively acting disease-alleles. Clues that tumour susceptibility may have a recessive basis come from reports of an increased incidence associated with consanguinity and in populations characterised by a high degree of inbreeding^11^,^12^,^13^,^14^,^15^,^16^. Further evidence for the role of homozygosity in cancer predisposition is provided by experimental animal inbreeding (*e.g*. backcrossing mice) increasing tumour incidence^17^. Specific situations of homozygosity have also been directly associated with cancer such as uniparental disomy through altered imprinting^18^.

Common regions of homozygosity (ROH), the result of autozygosity (*i.e*. the occurrence of two alleles at the same locus originating from a common ancestor by way of non-random mating), have been shown to occur at high frequency in outbred populations as a result of selection^19^. Searching for ROH on a genome-wide basis therefore provides a strategy for exposing recessively acting disease genes. Recently Yang *et al*. studied patients with rheumatoid arthritis (RA) of Northern/Western European ancestry by whole-genome loss of heterozygosity analysis using Affymetrix SNP arrays^20^. A significant increase in the frequency of homozygosity was shown in cases compared with controls in the HLA region, a locus known to be associated with RA risk. Simón-Sánchez *et al*. showed that cases of early onset Parkinson’s disease harboured significantly more homozygous regions than healthy individuals^21^. Findings from these studies support the hypothesis that there exist multiple, recessive, disease-predisposing loci, which are not readily detected using a conventional GWAS approach based on analysis of individual SNPs. A possible explanation for this is that relative risks per locus are too low and/or that the disease-associated variants are not in strong linkage disequilibrium (LD) with tagSNPs, perhaps because of low allele frequencies.

While GWAS have limited ability to identify recessive disease alleles through single SNP analyses, these datasets can potentially be exploited to search for recessively acting disease loci through whole genome homozygosity analysis (WGHA). Hence to examine whether homozygosity is associated with an increased risk of developing HL and to search for novel recessively-acting disease loci, we conducted a WGHA of 589 HL cases and 5,199 controls genotyped for 484,072 tagging SNPs^4^.

## Study Design

### Patients and DNA samples

We analysed the constitutional DNA of 622 patients with HL ascertained from the UK. Details of the study are provided in previously published material^4^.

### Control series

We used data from two publicly accessible data series for population SNP genotype frequencies: individuals from the UK Welcome Trust Case-Control Consortium 2 (WTCCC2) study of 2,930 individuals from the 1958 British Birth Cohort (58C; also known as the National Child Development Study)^22^ and 2,737 individuals from the UK Blood Service collections (UKBS), all of which were combined to provide genotype data from 5,667 controls.

### Ethics

Collection of blood samples and clinico-pathological information from subjects was undertaken with informed consent and relevant ethical review board approval (the Royal Marsden Hospital NHS Trust and Multicentre Research Ethics Committee) in accordance with the tenets of the Declaration of Helsinki.

### Genotyping

As previously described^4^, DNA from the cases was extracted and quantified from EDTA-venous blood samples using conventional methodologies and a genome-wide scan of tagging SNPs conducted using Illumina Infinium 660w-Quad Bead Chips according to the manufacturer’s protocols (Illumina, San Diego, USA). The control series had already been genotyped using the Illumina Human1.2M-Duo Custom_v1 Array BeadChips. We restricted our analysis to the autosomal SNPs. We considered that a DNA sample had failed if it did not generate a genotype for >95% of loci. Similarly, a SNP was considered a failure if <95% of DNA samples generated a genotype at the locus. To ensure quality of genotyping, a series of duplicate samples were genotyped on the same arrays, with concordance rates of >99.9%. The overall genotyping call rate was 99.92%.

### Quality control

To identify samples showing relatedness, identity by state values (IBS) were calculated for pairs of individuals. For any pair with >80% identical SNP genotypes, we removed the sample with the lower call rate from the analysis. We excluded SNPs on the basis of deviation from Hardy-Weinberg Equilibrium (HWE) using a threshold of *P* < 1 × 10^−5^ in either the cases or controls, which resulted in only 139 SNPs being removed from the analysis. We also removed SNPs with MAF <0.05. To identify and exclude individuals with non-Western European ancestry, case and control data were merged with data obtained from individuals of different ethnicities from the International HapMap Project, from genome-wide IBS distances for markers shared between HapMap and our SNP panel, and from dissimilarity measures used to perform principal component analysis. After imposing these stringent quality control measures, 484,072 SNP genotypes were available on 589 HL cases and 5,199 controls which formed the basis of our analysis.

### Statistical and bioinformatics analysis

We detected ROH using PLINK v1.90^23^, which moves a sliding window of SNPs across the entire genome. To allow for genotyping error or other sources of artificial heterozygosity (such as paralogous sequences) within a stretch of truly homozygous SNPs, 2% heterozygous SNPs were allowed in each window. This was done to prevent underestimation of the number and size of ROH. We left the remaining options set to the default values (including allowing 5 missing calls per window), except that we varied the parameter homozyg-snp according to our heuristic preferences for defining the ROH as detailed below. Subsequent statistical analyses were performed using packages available in R (version 3.1.2) and specifically written Perl code. Comparison of the distribution of categorical variables was performed using the χ^2^ test. To compare the difference in average number of ROH between cases and controls we used the Student t-test. Naïve adjustment for multiple testing was based on the Bonferroni correction. In addition to conducting a PLINK-based analysis of ROH we also subsequently analysed our data using SNP & Variation Suite 8 software (Golden Helix, Bozeman, MT, USA) which is based on implementation of a fixed window.

We used three metrics to investigate the selection pressure on each ROH. Integrated Haplotype Score (iHS) is based on LD surrounding a positively selected allele compared to background, providing evidence of recent positive selection at a locus^24^. An iHS score >2.0 reflects that haplotypes on the ancestral background are longer compared to the derived allelic background. Episodes of selection tend to skew SNP frequencies in different directions and Tajima’s D is based on the frequencies of SNPs segregating in the region of interest^25^. Fixation index (F_st_) measures the degree of population differentiation at a locus, taking values from 0 to 1.0^26^. iHS, D and F_st_ metrics were obtained from dbPSHP^27^.

### Identification of ROH

In order to focus on commonly occurring ROH and to empower our analysis to identify meaningful associations, only ROH in which 10 or more individuals shared the same ROH were retained for analysis. The initial search for ROH was performed using PLINK^23^ with a specified length of 84 consecutive SNPs (homozyg-snp parameter). This ROH length was chosen to be more than an order of magnitude larger than the mean haploblock size in the human genome without being so large as to be very rare. The likelihood of observing 84 consecutive chance events can be calculated as follows^19^. Mean heterozygosity in the controls was calculated to be 34%. Thus, given 484,072 SNPs and 5,788 individuals, a minimum length of 60 would be required to produce <5% randomly generated ROH across all subjects ([1 − 0.34]^60^ × 484,072 × 5788 = 0.043). A consequence of LD is that the SNP genotypes are not always independent, thereby inflating the probability of chance occurrences of biologically meaningless ROH. Analysis based on PLINK’s pairwise LD SNP pruning function showed 363,152 separable tag groups, representing a 25% reduction of information compared to the original number of SNPs. Thus ROH of length 84 were used to approximate the degrees of freedom of 60 independent SNP calls.

Once all ROH of at least 84 SNPs in length were identified, these were pruned to only those ROH that occurred in more than 10 individuals. To ensure that a minimum length and minimum number of SNPs in each ROH was maintained, each individual’s SNP data were recoded as one if the SNP was in an ROH for that individual and zero otherwise. Then, for each SNP, those SNPs with fewer than 10 individuals coded as one were recoded to zero before removing any ROH that due to this recoding were now less than the required number of SNPs in length. This process therefore resulted in a list of “common” ROH having a minimum of 75 consecutive ROH calls across 10 or more samples and with each ROH having the same start and end locations across all individuals where that ROH is observed.

## Results

**Figure 1:**
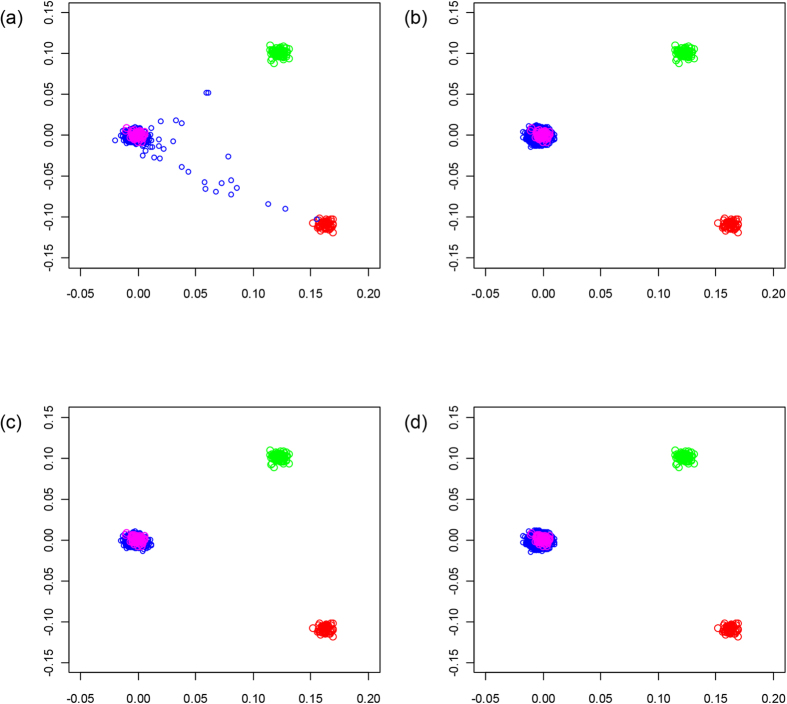
Identification of individuals in the GWAS of non-European ancestry. The first two principal components of the analysis were plotted. HapMap CEU individuals are plotted in blue; CHB + JPT individuals are plotted in green; YRI individuals are plotted in red; GWAS cases are plotted in pink before (**a**) and after (**c**) removal, GWAS controls are plotted pink before (**b**) and after (**d**) removal.

We have previously subjected cases and controls to rigorous quality control in terms of excluding samples and SNPs with poor call rates. Furthermore, we excluded SNPs showing significant departure from HWE. The results in Tables 1 and 2 were not substantively different when including the additional 139 SNPs that failed the HWE threshold of *P* < 1 × 10^−5^. We evaluated datasets for ancestral differences by principal component analysis and removed all outliers. Figure 1 shows that the final sample series used were ancestrally comparable and hence could be pooled without introducing systematic bias.

**Figure 2:**
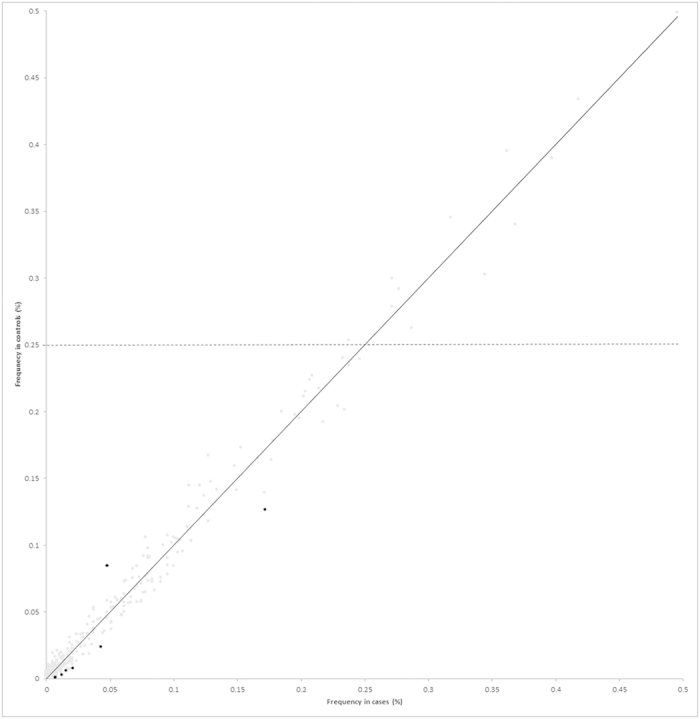
Comparison between the frequencies of the 398 ROH identified, in HL cases and the controls. The seven ROH coloured black are those that are significantly associated with HL risk (P < 0.01) and the horizontal line shows ROH with frequency >25% in controls.

**Figure 3:**
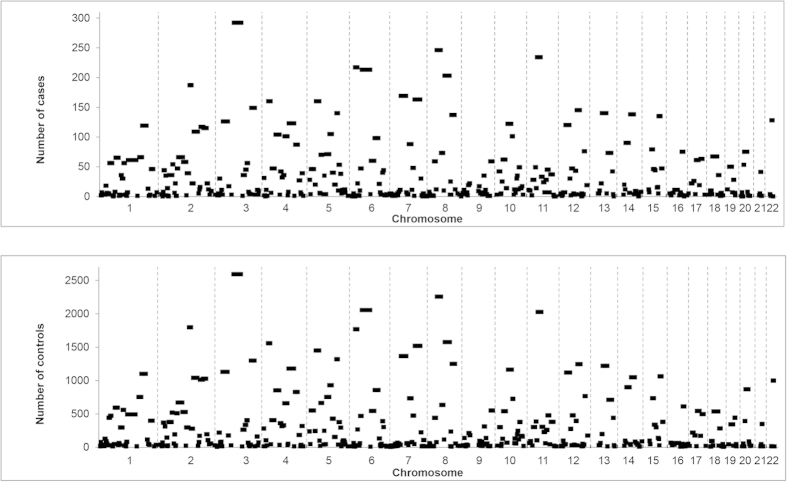
Plots showing the location of the ROH identified in HL cases and controls. The location of the ROH in the genomes of the cases is shown in (**A**) and the controls (**B**).

Using PLINK a total of 398 common ROH were identified in samples (Supplementary Table 1), encompassing approximately 60% of the autosome as measured by total chromosomal length. Figure 2 shows correlation between the frequency of individual ROH in the cases and the controls, while Fig. 3 shows the similarity between the genome-wide plots of the location of each ROH among the genomes of HL cases and controls. We repeated our ROH analysis of cases and controls using SNP & Variation Suite Software. Our findings were not materially affected using this alternative approach; hence we confined our further analysis to PLINK-based methodology.

The 37 longest ROH exceeded 12 Mb in length each and included ROH encompassing the centromeric regions of chromosomes 1, 2, 3, 4, 5, 6, 7, 8, 11, 12, 16, and 20. The lengths of these ROH are partly a consequence of long regions for which there are no annotating SNPs. This is however unlikely to be the sole explanation, as in each case these centromeric regions were flanked by large homozygous regions containing numerous SNPs. One of these centromeric regions (chromosome 8) has been previously highlighted in several genome-wide studies of selective sweeps, thus providing validation of our methodology^24^,^28^,^29^,^30^. Twenty-five non-centromeric regions harbouring ROH greater than 12 Mb in length were identified in our study (Supplementary Table 1).

The ROH covering the largest genomic region (40 Mb) and the largest number of SNPs (2885) was found to be ROH83 spanning the centromere of chromosome 3, a region previously shown to be characterised by a high frequency of ROH in the European population^28^.

There are twelve ROH that were very common (>25% frequency) in the control series (Table 1). Five of these are included in the nine most common ROH found in Lencz *et al*.^19^ and harbour several gene categories identified in various studies that appear to be influenced by a high degree of selective pressure^24^,^28^,^29^,^30^. Publicly available data from HapMap and the WTCCC do not indicate that these regions have excessive copy number variation or segmental duplication, nor do they have very low recombination rates^31^. However, the high iHS, D and F_st_ metrics for each region are compatible with positive selection in Caucasian populations (Table 1).

**Figure 4:**
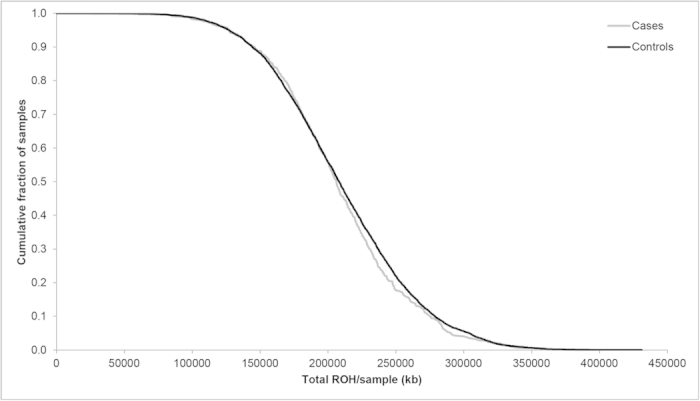
Cumulative distributions of ROH in HL cases and controls. The graph is presented in such a way that each data point represents the cumulative fraction (y-axis) of the samples with the corresponding minimum cumulative run of homozygosity (x-axis).

The total number of common ROH observed in each individual was calculated to permit genome-wide comparison between the case and control groups. Each individual ROH therefore was assigned a value between 0 and 398. Overall, patients with HL (mean = 17.7, SD = 4.6) and controls (mean = 17.8, SD = 4.0) showed no significant difference in the average number of ROH (t_5788_ = 0.2851, P = 0.76). To also examine whether there were differences in the distributions of ROH in the genomes of cases and controls we computed the cumulative distributions of both series (Fig. 4). This analysis also provides no support for a difference in autozygosity profiles between cases and controls on a genome-wide basis.

At an individual level seven ROH, none of which include an excessive number of copy number variants, differed significantly (*P* < 0.01) between cases and controls (Table 2). Adjusting for age and sex did not yield a significant difference in results. While these associations were not individually statistically significant after adjusting for multiple testing using the Bonferroni correction, imposing such adjustment is highly conservative and can lead to type 2 error. Six of these seven, marginally significant, ROH were more common in the controls than in the cases.

ROH111 was identified in 17.1% of cases (n = 101) compared with 12.7% of controls (n = 660) (*P* = 0.0017). Over 50 genes or predicted transcripts map to the region encompassed by this ROH including the gene encoding NF-kappa-B p105 subunit (*NFKB1*; MIM 164011). Although speculative, it is intriguing to note that NF-κB pathways are constitutively overactive in a variety of lymphomas including HL^32^,^33^. Mutations in *NFKB1* have been identified in tumour samples from patients with HL^34^, and c-Rel encoded by *REL* (a known causative locus in HL) interacts with NF-kappa-B p105 subunit as a mediator of immune and inflammatory responses^4^,^35^. We explored the possibility of a relationship between *NFKB1* genotype and HL risk through single point analysis based on SNPs that map within 25 kb of the gene. The strongest association was provided by an imputed SNP located in an intron of *NFKB1*, rs4647965 (OR = 1.9, *P*_*cohrane*−*armitage*_ = 0.0004, info = 0.79).

## Discussion

Recent studies have provided evidence that signatures of autozygosity correlate to cancer incidence and that these regions showing identity by descent may be the locations of genes contributing to tumour heritability^36^,^37^ as well as other diseases^20^,^21^. These data have been interpreted as providing an explanation for the increased cancer rates reported in some inbred populations^11^,^13^,^14^.

Here we have used a high-density genomic scan to compare the structure of genetic variation in patients with HL with healthy controls. This same sample series has recently been used to identify six predisposition loci for HL^4^,^8^. By imposing stringent quality control we have ensured individuals in our study were from an apparently panmictic population (i.e. population where all individuals are potential partners) with no evidence of stratification. Our data provide further evidence that ROH, ranging in size from 6 to 40 Mb, are common in individuals from an outbred population^38^,^39^,^40^,^41^. As documented in Table 1, the common ROH we identified are representative of autozygosity due to distant consanguinity and not chromosomal abnormalities or common copy number variants. Moreover these homozygous regions are too common and small to be a consequence of recent consanguinity and are consistent with the possibility that they mark regions under selective pressure^42^. Based on our analysis there was no evidence for an association between homozygosity and HL risk on the basis of total ROH size per individual. While not formally statistically significant after adjustment for multiple testing, the associations between HL risk and ROH, as demonstrated by ROH111 (which harbours *NF-κB1*), and ROH375 (previously implicated in susceptibility to childhood B-cell precursor acute lymphoblastic leukaemia [BCP-ALL])^43^, may reflect regions that warrant further investigation.

The assertion that increased autozygosity correlates with cancer incidence provides an attractive explanation for reported increased cancer risk in inbred populations. However, as recently articulated, several criticisms can be levelled at such an idea^44^. The observation of an increased cancer risk associated with consanguinity has often been based on studies of a small number of individuals in an isolated community or a single large family with a high level of inbreeding. Thus, the relevance of inbreeding to the population risk of cancer is unclear, as inbreeding and founder effects may be confounded. Sample sizes in the molecular studies^36^,^37^, which have sought to establish a relationship between ROH and cancer risk have generally been small and, crucially, case and control groups ethnically heterogeneous or unmatched. Here we have addressed these possible shortcomings in our study of HL by analysing a large set of cases and controls that have been genotyped for several hundred thousand SNPs and imposed a high level of quality control both in terms of genotyping and sample ancestry. In addition, WGHA in breast, prostate and colorectal cancer as well as childhood BCP-ALL have found limited evidence that measured homozygosity confers an increased risk of developing these cancers^43^,^44^,^45^.

In conclusion our findings make it unlikely that levels of measured common homozygosity, from autozygosity, uniparental isodisomy or hemizygosity, play a significant role in defining the risk of developing HL in a predominantly outbred population. Moreover it is unlikely that there exist large numbers of recessive alleles in most European populations, that predispose to HL and are unmasked by autozygosity. This analysis does not however exclude the possibility that recessively acting disease alleles exist for HL.

## Additional Information

**How to cite this article**: Sud, A. *et al*. Genome-wide homozygosity signature and risk of Hodgkin lymphoma. *Sci. Rep*. **5**, 14315; doi: 10.1038/srep14315 (2015).

## Supplementary Material

Supplementary Information

## Figures and Tables

**Table 1:** List of ROH with frequency of >25% in the controls.

ROH	Chromosome	Start, bp	End, bp	Length, bp	No. of SNPs	% of controls	iHS_max_	Tajima D_max_	Fst_max_
ROH83	3	74364811	115347556	40982745	4861	50	3.30	4.27	0.52
ROH212	8	41237437	58695807	17458370	1660	43	3.00	3.99	0.40
ROH159	6	53470082	88671201	35201119	4749	40	3.36	4.00	0.43
ROH272	11	44985936	60832282	15846346	1379	39	2.52	3.93	0.80
ROH60	2	133966177	142500134	8533957	1532	35	3.10	4.25	0.56
ROH153	6	24729115	33874440	9145325	2439	34	4.18	4.59	0.51
ROH215	8	74146766	96245252	22098486	3065	30	2.87	4.45	0.33
ROH103	4	27284946	37133279	9848333	1366	30	2.78	4.59	0.43
ROH196	7	106400535	130396888	23996353	3310	29	3.55	4.63	0.42
ROH126	5	39081475	53519724	14438249	1551	28	2.47	3.78	0.38
ROH188	7	47907214	70407537	22500323	2518	26	3.02	4.50	0.70
ROH137	5	125886071	132587049	6700978	882	25	2.78	4.10	0.31

Chromosomal coordinates derived from the National Center for Biotechnology Information (NCBI) build 36.

*Represent maximal values for several metrics of positive selection, derived from dbpSHP^27^.

**Table 2:** List of ROH associated with HL risk (*P* < 0.01).

ROH	Chromosome	Start, bp	End, bp	Length, bp	No. of SNPs	No. (%) of cases	No. (%) of controls	Χ2	P
ROH118	4	164181835	165694435	1512600	280	7 (1.18)	16 (0.31)	10.47	0.0012
ROH111	4	96397988	109621884	13223896	1816	101 (17.15)	660 (12.70)	9.86	0.0017
ROH174	7	3362918	4133399	770481	196	4 (0.68)	6 (0.12)	9.81	0.0017
ROH378	19	36097328	39542089	3444761	355	28 (4.75)	442 (8.50)	9.53	0.0020
ROH375	19	9314135	12772090	3457955	412	12 (2.04)	43 (0.83)	8.39	0.0038
ROH187	7	43086042	46983232	3897190	648	11 (1.87)	38 (0.73)	8.29	0.0040
ROH251	10	30830837	33710224	2879387	483	25 (4.24)	126 (2.42)	7.15	0.0075
