# Supplementary material for: Genome-wide homozygosity signature and risk of Hodgkin lymphoma
Source: Sci Rep. 2015 Sep 22;5:14315. doi: 10.1038/srep14315 (PMC4585760; doi:10.1038/srep14315)
Supplement: Supplementary Information [file srep14315-s1.doc]

**SUPPLEMENTARY INFORMATION**

**Genome-wide homozygosity signature and risk of Hodgkin lymphoma**

Amit Sud, Rosie Cooke, Anthony J Swerdlow, Richard S Houlston

| ROH ID | Num. of cases | Num. of controls | *P* Value | Chromosome | Range (SNPs) | Range (bp) | Num. of SNPs | Length |
| --- | --- | --- | --- | --- | --- | --- | --- | --- |
| ROH1 | 2 | 19 | 0.923 | 1 | rs3789553 - rs11120889 | 6117171 - 7324323 | 227 | 1207152 |
| ROH2 | 2 | 13 | 0.674 | 1 | rs9434498 - rs12091891 | 7711758 - 9000220 | 189 | 1288462 |
| ROH3 | 7 | 77 | 0.590 | 1 | rs17393334 - rs776909 | 9432911 - 10845821 | 171 | 1412910 |
| ROH4 | 4 | 39 | 0.856 | 1 | rs736185 - rs2977272 | 15705072 - 17536952 | 189 | 1831880 |
| ROH5 | 3 | 19 | 0.577 | 1 | rs97922 - rs2010397 | 20804065 - 22141983 | 250 | 1337918 |
| ROH6 | 3 | 17 | 0.464 | 1 | rs6670405 - rs10794665 | 23189123 - 24446128 | 127 | 1257005 |
| ROH7 | 18 | 127 | 0.339 | 1 | rs742390 - rs4654347 | 25499030 - 29779305 | 416 | 4280275 |
| ROH8 | 6 | 88 | 0.232 | 1 | rs6695189 - rs6701268 | 31354077 - 34041191 | 280 | 2687114 |
| ROH9 | 4 | 54 | 0.420 | 1 | rs2359106 - rs16823426 | 34962675 - 37193645 | 207 | 2230970 |
| ROH10 | 5 | 29 | 0.368 | 1 | rs9438989 - rs4660473 | 39372843 - 41318440 | 272 | 1945597 |
| ROH11 | 56 | 442 | 0.349 | 1 | rs4660192 - rs4926916 | 41842350 - 48085268 | 874 | 6242918 |
| ROH12 | 56 | 471 | 0.611 | 1 | rs560004 - rs1181183 | 48549673 - 54252198 | 585 | 5702525 |
| ROH13 | 2 | 33 | 0.392 | 1 | rs2495511 - rs11810204 | 55476178 - 57020516 | 348 | 1544338 |
| ROH14 | 0 | 14 | 0.212 | 1 | rs11207112 - rs942127 | 58279107 - 59284219 | 254 | 1005112 |
| ROH15 | 2 | 13 | 0.674 | 1 | rs2897708 - rs602476 | 60201195 - 61412781 | 233 | 1211586 |
| ROH16 | 8 | 48 | 0.293 | 1 | rs2806535 - rs1340589 | 64577770 - 67924391 | 660 | 3346621 |
| ROH17 | 65 | 592 | 0.741 | 1 | rs1367447 - rs12041774 | 68634030 - 81482945 | 2049 | 12848915 |
| ROH18 | 1 | 35 | 0.147 | 1 | rs563189 - rs315552 | 83284533 - 84795283 | 212 | 1510750 |
| ROH19 | 3 | 66 | 0.114 | 1 | rs17370490 - rs7518015 | 85389342 - 87826418 | 472 | 2437076 |
| ROH20 | 2 | 42 | 0.224 | 1 | rs3008445 - rs1215649 | 88446605 - 90278842 | 241 | 1832237 |
| ROH21 | 36 | 297 | 0.615 | 1 | rs4559484 - rs538880 | 90659140 - 94558774 | 624 | 3899634 |
| ROH22 | 30 | 296 | 0.569 | 1 | rs17111996 - rs1922987 | 95122518 - 101753101 | 1134 | 6630583 |
| ROH23 | 56 | 558 | 0.383 | 1 | rs1415105 - rs10494066 | 102315214 - 107789508 | 702 | 5474294 |
| ROH24 | 3 | 25 | 0.898 | 1 | rs656709 - rs608196 | 108073587 - 109808117 | 305 | 1734530 |
| ROH25 | 2 | 24 | 0.686 | 1 | rs351359 - rs4307568 | 113040277 - 114669775 | 225 | 1629498 |
| ROH26 | 4 | 39 | 0.856 | 1 | rs1994291 - rs12402914 | 114680913 - 116233410 | 274 | 1552497 |
| ROH27 | 1 | 11 | 0.840 | 1 | rs4348723 - rs7552721 | 116239633 - 116903246 | 95 | 663613 |
| ROH28 | 7 | 81 | 0.504 | 1 | rs3767784 - rs11249431 | 117564909 - 121286203 | 475 | 3721294 |
| ROH29 | 61 | 492 | 0.410 | 1 | rs11249395 - rs7532302 | 121311799 - 156659768 | 1030 | 35347969 |
| ROH30 | 1 | 13 | 0.716 | 1 | rs2774279 - rs2340727 | 161017556 - 161946727 | 161 | 929171 |
| ROH31 | 66 | 753 | 0.037 | 1 | rs1322489 - rs12022368 | 168960794 - 180934047 | 1844 | 11973253 |
| ROH32 | 119 | 1100 | 0.564 | 1 | rs10797724 - rs2816939 | 181518125 - 199985700 | 2488 | 18467575 |
| ROH33 | 13 | 117 | 0.897 | 1 | rs1928444 - rs12083980 | 205366268 - 208278877 | 430 | 2912609 |
| ROH34 | 1 | 48 | 0.062 | 1 | rs2236891 - rs1655996 | 209804499 - 213491399 | 638 | 3686900 |
| ROH35 | 3 | 38 | 0.557 | 1 | rs4604670 - rs3845529 | 214581484 - 216136620 | 277 | 1555136 |
| ROH36 | 46 | 398 | 0.753 | 1 | rs17047703 - rs496663 | 218525588 - 229098232 | 1455 | 10572644 |
| ROH37 | 1 | 14 | 0.662 | 1 | rs7531062 - rs16833075 | 235095183 - 236157277 | 108 | 1062094 |
| ROH38 | 7 | 38 | 0.221 | 1 | rs2343121 - rs2047137 | 242700803 - 244196410 | 206 | 1495607 |
| ROH39 | 4 | 41 | 0.786 | 2 | rs7590964 - rs11693212 | 13126278 - 15825003 | 393 | 2698725 |
| ROH40 | 3 | 30 | 0.845 | 2 | rs2341991 - rs876030 | 16990911 - 18544954 | 263 | 1554043 |
| ROH41 | 0 | 15 | 0.197 | 2 | rs4240224 - rs6721815 | 19012808 - 19854280 | 178 | 841472 |
| ROH42 | 8 | 73 | 0.909 | 2 | rs587139 - rs10206536 | 20948679 - 23155213 | 250 | 2206534 |
| ROH43 | 44 | 369 | 0.642 | 2 | rs2577756 - rs10186460 | 23601556 - 28789643 | 631 | 5188087 |
| ROH44 | 35 | 310 | 0.832 | 2 | rs12992405 - rs7575204 | 31397332 - 33645650 | 396 | 2248318 |
| ROH45 | 2 | 24 | 0.686 | 2 | rs1965391 - rs17017895 | 34237472 - 36175700 | 404 | 1938228 |
| ROH46 | 14 | 144 | 0.598 | 2 | rs1374190 - rs13429070 | 38146232 - 42085323 | 787 | 3939091 |
| ROH47 | 1 | 18 | 0.487 | 2 | rs11686998 - rs6738922 | 42188641 - 43418616 | 224 | 1229975 |
| ROH48 | 0 | 28 | 0.077 | 2 | rs17031289 - rs1657854 | 43922928 - 45210805 | 324 | 1287877 |
| ROH49 | 36 | 378 | 0.324 | 2 | rs11675417 - rs1553933 | 47754445 - 60509080 | 2620 | 12754635 |
| ROH50 | 54 | 519 | 0.545 | 2 | rs243035 - rs1421336 | 60601376 - 64702082 | 520 | 4100706 |
| ROH51 | 6 | 56 | 0.893 | 2 | rs10183139 - rs13389582 | 69148310 - 70838190 | 236 | 1689880 |
| ROH52 | 22 | 192 | 0.847 | 2 | rs2114811 - rs7564471 | 71932031 - 74965590 | 352 | 3033559 |
| ROH53 | 1 | 20 | 0.420 | 2 | rs10173399 - rs7589991 | 75721583 - 76947107 | 271 | 1225524 |
| ROH54 | 1 | 28 | 0.237 | 2 | rs11895211 - rs11893407 | 77798886 - 79193189 | 232 | 1394303 |
| ROH55 | 47 | 509 | 0.175 | 2 | rs17339856 - rs960066 | 80279831 - 85700924 | 676 | 5421093 |
| ROH56 | 66 | 670 | 0.268 | 2 | rs6750610 - rs7608291 | 85731858 - 105593028 | 1626 | 19861170 |
| ROH57 | 58 | 529 | 0.752 | 2 | rs9989768 - rs6724530 | 106081221 - 119789098 | 1970 | 13707877 |
| ROH58 | 2 | 23 | 0.729 | 2 | rs11674898 - rs11123550 | 119879640 - 121122730 | 151 | 1243090 |
| ROH59 | 39 | 297 | 0.327 | 2 | rs13015322 - rs1026892 | 121930158 - 131641471 | 1377 | 9711313 |
| ROH60 | 187 | 1795 | 0.190 | 2 | rs1836216 - rs6758126 | 133966177 - 142500134 | 1532 | 8533957 |
| ROH61 | 22 | 278 | 0.105 | 2 | rs10496893 - rs13024803 | 142505730 - 151077809 | 1125 | 8572079 |
| ROH62 | 109 | 1041 | 0.395 | 2 | rs16828256 - rs1806328 | 151347639 - 169506879 | 2769 | 18159240 |
| ROH63 | 5 | 22 | 0.145 | 2 | rs2270336 - rs7574002 | 171557049 - 173124183 | 219 | 1567134 |
| ROH64 | 12 | 77 | 0.281 | 2 | rs6433472 - rs12472052 | 175143309 - 177113656 | 289 | 1970347 |
| ROH65 | 16 | 172 | 0.463 | 2 | rs11679930 - rs1554261 | 177370744 - 180297960 | 529 | 2927216 |
| ROH66 | 117 | 1013 | 0.615 | 2 | rs951427 - rs12693598 | 180483057 - 192343237 | 1539 | 11860180 |
| ROH67 | 115 | 1027 | 0.704 | 2 | rs6434538 - rs2358495 | 192991640 - 206500903 | 1639 | 13509263 |
| ROH68 | 22 | 193 | 0.855 | 2 | rs4675571 - rs796211 | 206813228 - 211955897 | 763 | 5142669 |
| ROH69 | 12 | 106 | 0.899 | 2 | rs1159709 - rs4672768 | 212715410 - 216214124 | 648 | 3498714 |
| ROH70 | 3 | 43 | 0.423 | 2 | rs6755375 - rs1867536 | 218776372 - 220581322 | 198 | 1804950 |
| ROH71 | 9 | 57 | 0.333 | 2 | rs2395982 - rs10192769 | 224257311 - 227699918 | 544 | 3442607 |
| ROH72 | 3 | 29 | 0.886 | 2 | rs4676369 - rs6737791 | 241716305 - 242918203 | 142 | 1201898 |
| ROH73 | 2 | 8 | 0.298 | 3 | rs2255558 - rs2619508 | 9416832 - 10215950 | 94 | 799118 |
| ROH74 | 5 | 26 | 0.262 | 3 | rs2454505 - rs11712537 | 11497325 - 13052644 | 285 | 1555319 |
| ROH75 | 2 | 20 | 0.873 | 3 | rs10510431 - rs861478 | 14829166 - 16280076 | 275 | 1450910 |
| ROH76 | 30 | 219 | 0.285 | 3 | rs1529513 - rs9863026 | 16563006 - 20984501 | 655 | 4421495 |
| ROH77 | 5 | 72 | 0.295 | 3 | rs9310685 - rs7633774 | 22207033 - 23733192 | 267 | 1526159 |
| ROH78 | 11 | 80 | 0.515 | 3 | rs13070407 - rs7613869 | 25493178 - 28656674 | 497 | 3163496 |
| ROH79 | 3 | 16 | 0.408 | 3 | rs1019855 - rs9825159 | 30697661 - 31755406 | 203 | 1057745 |
| ROH80 | 126 | 1129 | 0.687 | 3 | rs347158 - rs12492799 | 32465430 - 54063875 | 2621 | 21598445 |
| ROH81 | 17 | 176 | 0.543 | 3 | rs193317 - rs10510813 | 55636745 - 59535975 | 655 | 3899230 |
| ROH82 | 3 | 27 | 0.951 | 3 | rs1491722 - rs7641148 | 68286023 - 69776724 | 336 | 1490701 |
| ROH83 | 292 | 2593 | 0.540 | 3 | rs529655 - rs13059137 | 74364811 - 115347556 | 4861 | 40982745 |
| ROH84 | 1 | 12 | 0.775 | 3 | rs2972469 - rs13078249 | 115448121 - 116468320 | 198 | 1020199 |
| ROH85 | 36 | 262 | 0.233 | 3 | rs1355718 - rs4679245 | 117086511 - 126184450 | 1619 | 9097939 |
| ROH86 | 45 | 335 | 0.230 | 3 | rs2001929 - rs6762405 | 127044373 - 133156692 | 869 | 6112319 |
| ROH87 | 56 | 405 | 0.120 | 3 | rs11915399 - rs10935348 | 133649908 - 139447500 | 837 | 5797592 |
| ROH88 | 8 | 69 | 0.892 | 3 | rs9822648 - rs4371486 | 139899766 - 142902953 | 484 | 3003187 |
| ROH89 | 5 | 39 | 0.768 | 3 | rs7616384 - rs9861465 | 143453195 - 145509215 | 272 | 2056020 |
| ROH90 | 4 | 24 | 0.458 | 3 | rs4318523 - rs2319333 | 145616862 - 147377697 | 277 | 1760835 |
| ROH91 | 4 | 15 | 0.113 | 3 | rs9864940 - rs13072463 | 147435803 - 148973465 | 265 | 1537662 |
| ROH92 | 4 | 40 | 0.821 | 3 | rs3105873 - rs9824588 | 150732589 - 153022780 | 376 | 2290191 |
| ROH93 | 149 | 1299 | 0.606 | 3 | rs4679705 - rs678690 | 153044220 - 168546914 | 2103 | 15502694 |
| ROH94 | 3 | 15 | 0.353 | 3 | rs2421748 - rs11924032 | 169366536 - 170735099 | 181 | 1368563 |
| ROH95 | 11 | 161 | 0.105 | 3 | rs4857739 - rs10937066 | 177472170 - 181557730 | 521 | 4085560 |
| ROH96 | 3 | 26 | 0.938 | 3 | rs1392340 - rs16858720 | 181659359 - 184066615 | 408 | 2407256 |
| ROH97 | 9 | 73 | 0.770 | 3 | rs4350902 - rs16848688 | 184352200 - 185848147 | 212 | 1495947 |
| ROH98 | 31 | 281 | 0.832 | 4 | rs747582 - rs16878083 | 8373832 - 11100788 | 427 | 2726956 |
| ROH99 | 2 | 16 | 0.877 | 4 | rs993835 - rs16902 | 11744388 - 13095942 | 217 | 1351554 |
| ROH100 | 0 | 18 | 0.157 | 4 | rs1316686 - rs11944611 | 14853491 - 15907133 | 181 | 1053642 |
| ROH101 | 11 | 105 | 0.809 | 4 | rs2518641 - rs11936122 | 17467715 - 20473278 | 394 | 3005563 |
| ROH102 | 1 | 27 | 0.254 | 4 | rs4285070 - rs7665116 | 22266680 - 23853011 | 258 | 1586331 |
| ROH103 | 160 | 1558 | 0.173 | 4 | rs1488276 - rs10031432 | 27284946 - 37133279 | 1366 | 9848333 |
| ROH104 | 6 | 43 | 0.608 | 4 | rs2011590 - rs9994274 | 38944101 - 40287396 | 202 | 1343295 |
| ROH105 | 47 | 407 | 0.754 | 4 | rs6447091 - rs1364989 | 41551813 - 55011769 | 1361 | 13459956 |
| ROH106 | 104 | 853 | 0.345 | 4 | rs6812462 - rs12508120 | 58085224 - 75650094 | 2171 | 17564870 |
| ROH107 | 3 | 35 | 0.654 | 4 | rs12501547 - rs4464594 | 75747264 - 77468785 | 299 | 1721521 |
| ROH108 | 42 | 361 | 0.742 | 4 | rs7667074 - rs12643434 | 77806097 - 83944060 | 870 | 6137963 |
| ROH109 | 32 | 316 | 0.552 | 4 | rs7696864 - rs2869929 | 84791189 - 89244007 | 698 | 4452818 |
| ROH110 | 36 | 329 | 0.801 | 4 | rs4399946 - rs6532503 | 90194997 - 95608023 | 709 | 5413026 |
| ROH111 | 101 | 660 | 0.002 | 4 | rs265057 - rs2077246 | 96397988 - 109621884 | 1816 | 13223896 |
| ROH112 | 2 | 34 | 0.368 | 4 | rs10009816 - rs7686686 | 109884269 - 111144505 | 208 | 1260236 |
| ROH113 | 8 | 54 | 0.455 | 4 | rs604612 - rs7675254 | 111466163 - 113825869 | 314 | 2359706 |
| ROH114 | 123 | 1180 | 0.332 | 4 | rs10008952 - rs7692053 | 114330615 - 137662604 | 3117 | 23331989 |
| ROH115 | 1 | 14 | 0.662 | 4 | rs1445523 - rs3843891 | 137828866 - 138609462 | 141 | 780596 |
| ROH116 | 87 | 829 | 0.471 | 4 | rs4627935 - rs3935569 | 141989050 - 153565838 | 1368 | 11576788 |
| ROH117 | 27 | 184 | 0.179 | 4 | rs17033585 - rs10009342 | 156735439 - 163179938 | 961 | 6444499 |
| ROH118 | 7 | 16 | 0.001 | 4 | rs11730501 - rs4692008 | 164181835 - 165694435 | 280 | 1512600 |
| ROH119 | 4 | 38 | 0.891 | 4 | rs9762579 - rs2018241 | 165877990 - 169340431 | 512 | 3462441 |
| ROH120 | 39 | 319 | 0.568 | 4 | rs973990 - rs11731252 | 169763252 - 174530927 | 588 | 4767675 |
| ROH121 | 1 | 14 | 0.662 | 4 | rs1479380 - rs4690665 | 175968691 - 177095908 | 138 | 1127217 |
| ROH122 | 28 | 238 | 0.756 | 5 | rs31781 - rs1502045 | 11529293 - 13745719 | 239 | 2216426 |
| ROH123 | 4 | 50 | 0.513 | 5 | rs398961 - rs40032 | 14824172 - 16508139 | 217 | 1683967 |
| ROH124 | 46 | 551 | 0.041 | 5 | rs2459826 - rs6872040 | 17453016 - 31075755 | 1827 | 13622739 |
| ROH125 | 20 | 243 | 0.172 | 5 | rs162909 - rs270592 | 34772786 - 38066586 | 590 | 3293800 |
| ROH126 | 160 | 1449 | 0.610 | 5 | rs10064513 - rs26771 | 39081475 - 53519724 | 1551 | 14438249 |
| ROH127 | 6 | 98 | 0.143 | 5 | rs11749226 - rs160078 | 53953264 - 55579789 | 275 | 1626525 |
| ROH128 | 5 | 21 | 0.121 | 5 | rs12518745 - rs159382 | 56881357 - 58172462 | 284 | 1291105 |
| ROH129 | 70 | 663 | 0.555 | 5 | rs17444059 - rs10076194 | 58560717 - 65600699 | 1039 | 7039982 |
| ROH130 | 4 | 41 | 0.786 | 5 | rs2125627 - rs4418079 | 71241513 - 72691120 | 209 | 1449607 |
| ROH131 | 35 | 248 | 0.186 | 5 | rs1039321 - rs4566770 | 73937506 - 75576313 | 250 | 1638807 |
| ROH132 | 4 | 46 | 0.623 | 5 | rs4704610 - rs7703068 | 79411248 - 82025104 | 516 | 2613856 |
| ROH133 | 71 | 752 | 0.128 | 5 | rs4282296 - rs7709763 | 83284724 - 95822590 | 1551 | 12537866 |
| ROH134 | 105 | 929 | 0.712 | 5 | rs897746 - rs2484103 | 95921529 - 106896617 | 1504 | 10975088 |
| ROH135 | 40 | 427 | 0.251 | 5 | rs35544 - rs152367 | 107102159 - 115189500 | 1468 | 8087341 |
| ROH136 | 20 | 155 | 0.534 | 5 | rs930922 - rs9687603 | 116375473 - 123223532 | 1153 | 6848059 |
| ROH137 | 140 | 1318 | 0.404 | 5 | rs1038381 - rs6890910 | 125886071 - 132587049 | 882 | 6700978 |
| ROH138 | 10 | 41 | 0.024 | 5 | rs6596146 - rs609385 | 133028493 - 134597267 | 254 | 1568774 |
| ROH139 | 53 | 377 | 0.105 | 5 | rs4246802 - rs10063472 | 135199709 - 141246646 | 765 | 6046937 |
| ROH140 | 38 | 293 | 0.370 | 5 | rs258765 - rs6874946 | 142574063 - 146545880 | 633 | 3971817 |
| ROH141 | 5 | 15 | 0.027 | 5 | rs4705159 - rs2053044 | 146678112 - 148205372 | 259 | 1527260 |
| ROH142 | 11 | 82 | 0.564 | 5 | rs998076 - rs10155614 | 150924769 - 153148731 | 398 | 2223962 |
| ROH143 | 7 | 102 | 0.202 | 5 | rs4958735 - rs2421050 | 153789590 - 158943044 | 898 | 5153454 |
| ROH144 | 12 | 81 | 0.359 | 5 | rs10062528 - rs10057965 | 160150822 - 163119808 | 493 | 2968986 |
| ROH145 | 6 | 42 | 0.572 | 5 | rs10068900 - rs17064971 | 163655400 - 165015242 | 209 | 1359842 |
| ROH146 | 1 | 13 | 0.716 | 5 | rs13174727 - rs11134996 | 175275485 - 176151747 | 96 | 876262 |
| ROH147 | 0 | 14 | 0.212 | 6 | rs9296384 - rs2165419 | 12463796 - 13422242 | 184 | 958446 |
| ROH148 | 2 | 14 | 0.745 | 6 | rs6914891 - rs9396642 | 15059299 - 16081474 | 204 | 1022175 |
| ROH149 | 5 | 66 | 0.394 | 6 | rs1745069 - rs6930954 | 17311516 - 19628633 | 413 | 2317117 |
| ROH150 | 0 | 12 | 0.248 | 6 | rs911361 - rs9348465 | 20415053 - 21330808 | 264 | 915755 |
| ROH151 | 1 | 9 | 0.972 | 6 | rs849877 - rs12529064 | 22298699 - 23190538 | 230 | 891839 |
| ROH152 | 0 | 11 | 0.269 | 6 | rs16888503 - rs1047782 | 24022898 - 24650731 | 184 | 627833 |
| ROH153 | 217 | 1768 | 0.112 | 6 | rs2328830 - rs17535965 | 24729115 - 33874440 | 2439 | 9145325 |
| ROH154 | 22 | 267 | 0.153 | 6 | rs2499762 - rs13202984 | 33948624 - 36581149 | 357 | 2632525 |
| ROH155 | 2 | 34 | 0.368 | 6 | rs6900617 - rs9462580 | 37652186 - 39815595 | 497 | 2163409 |
| ROH156 | 1 | 23 | 0.338 | 6 | rs9349232 - rs833069 | 42387426 - 43742579 | 176 | 1355153 |
| ROH157 | 10 | 59 | 0.220 | 6 | rs9349288 - rs10948262 | 44440781 - 45840604 | 244 | 1399823 |
| ROH158 | 47 | 470 | 0.415 | 6 | rs1209198 - rs9474312 | 46017626 - 52598501 | 1133 | 6580875 |
| ROH159 | 213 | 2055 | 0.123 | 6 | rs475333 - rs16879998 | 53470082 - 88671201 | 4749 | 35201119 |
| ROH160 | 4 | 9 | 0.013 | 6 | rs1325006 - rs9362636 | 89209992 - 90026797 | 157 | 816805 |
| ROH161 | 60 | 545 | 0.759 | 6 | rs1144171 - rs714654 | 91698265 - 106011016 | 2190 | 14312751 |
| ROH162 | 98 | 859 | 0.697 | 6 | rs7774958 - rs10872265 | 107493418 - 123708198 | 2503 | 16214780 |
| ROH163 | 21 | 133 | 0.136 | 6 | rs539794 - rs7760943 | 125416400 - 129869267 | 560 | 4452867 |
| ROH164 | 7 | 35 | 0.155 | 6 | rs4897350 - rs2255071 | 130284464 - 132880648 | 520 | 2596184 |
| ROH165 | 4 | 16 | 0.141 | 6 | rs8192624 - rs12193973 | 132892253 - 134151297 | 264 | 1259044 |
| ROH166 | 3 | 54 | 0.227 | 6 | rs7747509 - rs1342642 | 134998188 - 137323213 | 286 | 2325025 |
| ROH167 | 40 | 391 | 0.542 | 6 | rs6927712 - rs223607 | 139706222 - 143706420 | 534 | 4000198 |
| ROH168 | 44 | 303 | 0.095 | 6 | rs761626 - rs6570773 | 144125687 - 147017697 | 425 | 2892010 |
| ROH169 | 2 | 25 | 0.645 | 6 | rs9322158 - rs7769185 | 149242095 - 150451024 | 236 | 1208929 |
| ROH170 | 4 | 10 | 0.022 | 6 | rs9372014 - rs628389 | 157072815 - 158080876 | 112 | 1008061 |
| ROH171 | 2 | 17 | 0.934 | 6 | rs2770113 - rs9347286 | 158194819 - 159515360 | 259 | 1320541 |
| ROH172 | 3 | 19 | 0.577 | 6 | rs9457733 - rs3798953 | 160255243 - 161689816 | 323 | 1434573 |
| ROH173 | 5 | 14 | 0.019 | 7 | rs1949783 - rs4719619 | 1303439 - 2684992 | 182 | 1381553 |
| ROH174 | 4 | 6 | 0.002 | 7 | rs4343997 - rs12701401 | 3362918 - 4133399 | 196 | 770481 |
| ROH175 | 5 | 27 | 0.296 | 7 | rs6974647 - rs2280918 | 5411296 - 7119469 | 177 | 1708173 |
| ROH176 | 4 | 21 | 0.325 | 7 | rs320788 - rs10440995 | 9757755 - 11231727 | 277 | 1473972 |
| ROH177 | 1 | 9 | 0.972 | 7 | rs9639102 - rs10246960 | 11852635 - 12705413 | 249 | 852778 |
| ROH178 | 3 | 12 | 0.203 | 7 | rs1529893 - rs10242167 | 15507516 - 16552572 | 226 | 1045056 |
| ROH179 | 3 | 17 | 0.464 | 7 | rs1448649 - rs13241157 | 17571140 - 18860014 | 282 | 1288874 |
| ROH180 | 2 | 14 | 0.745 | 7 | rs2158495 - rs13244585 | 19021592 - 20122811 | 220 | 1101219 |
| ROH181 | 1 | 54 | 0.042 | 7 | rs886159 - rs11771214 | 22580985 - 24548327 | 403 | 1967342 |
| ROH182 | 3 | 45 | 0.378 | 7 | rs17151656 - rs849314 | 25496143 - 28282062 | 588 | 2785919 |
| ROH183 | 3 | 17 | 0.464 | 7 | rs7794697 - rs255121 | 29601142 - 30740576 | 169 | 1139434 |
| ROH184 | 5 | 70 | 0.325 | 7 | rs7781544 - rs16879245 | 32066091 - 33804485 | 314 | 1738394 |
| ROH185 | 2 | 24 | 0.686 | 7 | rs13223298 - rs2392415 | 34198270 - 36121541 | 328 | 1923271 |
| ROH186 | 8 | 56 | 0.513 | 7 | rs4576333 - rs7780802 | 38153390 - 41407459 | 583 | 3254069 |
| ROH187 | 11 | 38 | 0.004 | 7 | rs11772186 - rs17172793 | 43086042 - 46983232 | 648 | 3897190 |
| ROH188 | 169 | 1365 | 0.144 | 7 | rs10951933 - rs12113420 | 47907214 - 70407537 | 2518 | 22500323 |
| ROH189 | 7 | 58 | 0.835 | 7 | rs4236247 - rs4717850 | 70911123 - 73349788 | 250 | 2438665 |
| ROH190 | 3 | 62 | 0.143 | 7 | rs2267824 - rs12154832 | 73930411 - 78060486 | 450 | 4130075 |
| ROH191 | 4 | 34 | 0.907 | 7 | rs508990 - rs10274707 | 78504119 - 79780292 | 180 | 1276173 |
| ROH192 | 88 | 733 | 0.467 | 7 | rs1034784 - rs9792007 | 82224180 - 93050980 | 1643 | 10826800 |
| ROH193 | 11 | 77 | 0.443 | 7 | rs6972935 - rs10486859 | 93583867 - 96575827 | 509 | 2991960 |
| ROH194 | 48 | 473 | 0.467 | 7 | rs6465598 - rs17157475 | 97225761 - 103580446 | 782 | 6354685 |
| ROH195 | 1 | 49 | 0.058 | 7 | rs4265124 - rs557657 | 103870154 - 105399126 | 291 | 1528972 |
| ROH196 | 163 | 1518 | 0.428 | 7 | rs4517053 - rs7785403 | 106400535 - 130396888 | 3310 | 23996353 |
| ROH197 | 30 | 223 | 0.328 | 7 | rs17166360 - rs12666242 | 132016733 - 134945250 | 445 | 2928517 |
| ROH198 | 2 | 14 | 0.745 | 7 | rs7811360 - rs834436 | 135960799 - 137260639 | 207 | 1299840 |
| ROH199 | 0 | 20 | 0.136 | 7 | rs6464696 - rs10246939 | 140042556 - 141672604 | 216 | 1630048 |
| ROH200 | 5 | 108 | 0.045 | 7 | rs5012030 - rs1404707 | 141942895 - 146984783 | 707 | 5041888 |
| ROH201 | 1 | 11 | 0.840 | 7 | rs12703055 - rs2302481 | 149582906 - 150488468 | 98 | 905562 |
| ROH202 | 0 | 14 | 0.212 | 7 | rs1986588 - rs4143342 | 158035784 - 159042351 | 153 | 1006567 |
| ROH203 | 8 | 62 | 0.696 | 8 | rs332022 - rs4275198 | 8783465 - 10485822 | 506 | 1702357 |
| ROH204 | 4 | 47 | 0.594 | 8 | rs17799348 - rs1044011 | 11333521 - 12941709 | 282 | 1608188 |
| ROH205 | 1 | 12 | 0.775 | 8 | rs4582565 - rs13278338 | 14878724 - 15224160 | 190 | 345436 |
| ROH206 | 1 | 12 | 0.775 | 8 | rs1346593 - rs17582024 | 15402936 - 16175495 | 259 | 772559 |
| ROH207 | 2 | 18 | 0.960 | 8 | rs7832345 - rs2976411 | 23624725 - 24861815 | 181 | 1237090 |
| ROH208 | 1 | 14 | 0.662 | 8 | rs7835853 - rs11783717 | 26662246 - 27856062 | 341 | 1193816 |
| ROH209 | 1 | 22 | 0.363 | 8 | rs10503827 - rs4732956 | 28167726 - 29339824 | 175 | 1172098 |
| ROH210 | 59 | 440 | 0.169 | 8 | rs7465109 - rs2275959 | 29680367 - 37455059 | 915 | 7774692 |
| ROH211 | 12 | 74 | 0.228 | 8 | rs10955035 - rs7821626 | 37738616 - 40865649 | 464 | 3127033 |
| ROH212 | 246 | 2255 | 0.406 | 8 | rs2883470 - rs1369776 | 41237437 - 58695807 | 1660 | 17458370 |
| ROH213 | 73 | 635 | 0.712 | 8 | rs17292859 - rs13275245 | 59736227 - 68916784 | 1284 | 9180557 |
| ROH214 | 10 | 117 | 0.404 | 8 | rs1873547 - rs10092844 | 70724911 - 72213290 | 223 | 1488379 |
| ROH215 | 203 | 1575 | 0.024 | 8 | rs2605877 - rs2439630 | 74146766 - 96245252 | 3065 | 22098486 |
| ROH216 | 3 | 17 | 0.464 | 8 | rs2439525 - rs11783750 | 97537041 - 98796043 | 193 | 1259002 |
| ROH217 | 25 | 235 | 0.760 | 8 | rs2443573 - rs2154637 | 99189845 - 101486501 | 221 | 2296656 |
| ROH218 | 3 | 9 | 0.087 | 8 | rs666026 - rs7818547 | 102557218 - 103529109 | 202 | 971891 |
| ROH219 | 137 | 1249 | 0.610 | 8 | rs1865336 - rs720131 | 103793370 - 117916015 | 2104 | 14122645 |
| ROH220 | 12 | 57 | 0.043 | 8 | rs10113582 - rs7005061 | 118808893 - 121781740 | 583 | 2972847 |
| ROH221 | 5 | 24 | 0.200 | 8 | rs4733852 - rs2719206 | 129269193 - 130408584 | 200 | 1139391 |
| ROH222 | 3 | 20 | 0.634 | 8 | rs7822138 - rs10101972 | 137343147 - 138616763 | 274 | 1273616 |
| ROH223 | 0 | 22 | 0.118 | 8 | rs901764 - rs2958492 | 144475424 - 145919126 | 143 | 1443702 |
| ROH224 | 6 | 33 | 0.270 | 9 | rs822349 - rs11794756 | 5442487 - 6785243 | 261 | 1342756 |
| ROH225 | 11 | 140 | 0.249 | 9 | rs10809144 - rs4740523 | 10647333 - 12453240 | 387 | 1805907 |
| ROH226 | 4 | 11 | 0.033 | 9 | rs10810590 - rs2383024 | 16689291 - 17487945 | 184 | 798654 |
| ROH227 | 7 | 40 | 0.271 | 9 | rs882123 - rs958562 | 20289153 - 22987003 | 554 | 2697850 |
| ROH228 | 7 | 26 | 0.034 | 9 | rs983967 - rs1758734 | 23608791 - 25263918 | 276 | 1655127 |
| ROH229 | 2 | 28 | 0.535 | 9 | rs836952 - rs1555453 | 25904637 - 27326780 | 360 | 1422143 |
| ROH230 | 19 | 212 | 0.337 | 9 | rs10968521 - rs10813841 | 28340971 - 32619211 | 779 | 4278240 |
| ROH231 | 22 | 181 | 0.688 | 9 | rs7038463 - rs2250340 | 33186156 - 36629417 | 479 | 3443261 |
| ROH232 | 9 | 33 | 0.015 | 9 | rs10781329 - rs4744632 | 71618245 - 74019486 | 488 | 2401241 |
| ROH233 | 17 | 95 | 0.070 | 9 | rs12341685 - rs12341208 | 74321381 - 77806643 | 553 | 3485262 |
| ROH234 | 5 | 21 | 0.121 | 9 | rs4361823 - rs1757948 | 79707908 - 81310680 | 276 | 1602772 |
| ROH235 | 7 | 83 | 0.465 | 9 | rs155531 - rs10780664 | 82535584 - 86960060 | 902 | 4424476 |
| ROH236 | 3 | 19 | 0.577 | 9 | rs329209 - rs10780767 | 87808147 - 89162323 | 214 | 1354176 |
| ROH237 | 35 | 310 | 0.832 | 9 | rs10820828 - rs7854146 | 93834060 - 98538954 | 685 | 4704894 |
| ROH238 | 7 | 55 | 0.740 | 9 | rs12551127 - rs925487 | 98921816 - 100636398 | 206 | 1714582 |
| ROH239 | 11 | 139 | 0.259 | 9 | rs1555521 - rs13284177 | 101687361 - 104093054 | 334 | 2405693 |
| ROH240 | 4 | 41 | 0.786 | 9 | rs10760891 - rs2777795 | 105173098 - 107672365 | 593 | 2499267 |
| ROH241 | 4 | 58 | 0.342 | 9 | rs2027401 - rs16925523 | 107742974 - 109267590 | 255 | 1524616 |
| ROH242 | 1 | 17 | 0.525 | 9 | rs12380632 - rs4978791 | 110941095 - 112040717 | 303 | 1099622 |
| ROH243 | 1 | 21 | 0.390 | 9 | rs10980679 - rs4979128 | 113747630 - 115289605 | 300 | 1541975 |
| ROH244 | 1 | 16 | 0.567 | 9 | rs10123153 - rs4145479 | 117728895 - 118838474 | 265 | 1109579 |
| ROH245 | 1 | 21 | 0.390 | 9 | rs1416940 - rs10513342 | 120133394 - 121482333 | 315 | 1348939 |
| ROH246 | 59 | 550 | 0.665 | 9 | rs10818407 - rs7850090 | 122696635 - 132085618 | 1391 | 9388983 |
| ROH247 | 3 | 26 | 0.938 | 10 | rs17144396 - rs12255818 | 8773922 - 10162933 | 254 | 1389011 |
| ROH248 | 42 | 300 | 0.159 | 10 | rs7080366 - rs11013962 | 17254832 - 24495586 | 1376 | 7240754 |
| ROH249 | 4 | 19 | 0.244 | 10 | rs10828658 - rs3006783 | 24776367 - 26673486 | 367 | 1897119 |
| ROH250 | 5 | 52 | 0.738 | 10 | rs11015149 - rs2993987 | 26812175 - 28800170 | 358 | 1987995 |
| ROH251 | 25 | 126 | 0.007 | 10 | rs7894785 - rs2804473 | 30830837 - 33710224 | 483 | 2879387 |
| ROH252 | 62 | 539 | 0.732 | 10 | rs7091026 - rs7091141 | 34219485 - 46148326 | 1095 | 11928841 |
| ROH253 | 14 | 69 | 0.039 | 10 | rs4342964 - rs10740244 | 48318619 - 53144956 | 685 | 4826337 |
| ROH254 | 122 | 1163 | 0.371 | 10 | rs11003565 - rs16926244 | 55076723 - 71082214 | 2832 | 16005491 |
| ROH255 | 101 | 725 | 0.026 | 10 | rs16929266 - rs17435206 | 73487127 - 77671322 | 426 | 4184195 |
| ROH256 | 3 | 60 | 0.161 | 10 | rs7915975 - rs4934394 | 81166039 - 82414710 | 185 | 1248671 |
| ROH257 | 12 | 131 | 0.494 | 10 | rs10509421 - rs4934150 | 82743607 - 87865374 | 954 | 5121767 |
| ROH258 | 6 | 57 | 0.867 | 10 | rs1870168 - rs303499 | 87940997 - 90553051 | 442 | 2612054 |
| ROH259 | 30 | 191 | 0.078 | 10 | rs7088963 - rs7913298 | 92905682 - 95233375 | 325 | 2327693 |
| ROH260 | 35 | 246 | 0.171 | 10 | rs10509665 - rs11595566 | 95528442 - 98436824 | 502 | 2908382 |
| ROH261 | 12 | 119 | 0.712 | 10 | rs2862297 - rs17113301 | 100081757 - 102396758 | 395 | 2315001 |
| ROH262 | 49 | 378 | 0.307 | 10 | rs10786609 - rs2245123 | 102635485 - 108553214 | 826 | 5917729 |
| ROH263 | 14 | 173 | 0.232 | 10 | rs17195022 - rs4918756 | 108811337 - 114267234 | 911 | 5455897 |
| ROH264 | 17 | 160 | 0.799 | 10 | rs1468500 - rs2530343 | 116057769 - 117851318 | 258 | 1793549 |
| ROH265 | 3 | 19 | 0.577 | 10 | rs1577074 - rs11199183 | 119983056 - 121901799 | 395 | 1918743 |
| ROH266 | 11 | 66 | 0.216 | 11 | rs4758059 - rs10840450 | 8338357 - 10653426 | 382 | 2315069 |
| ROH267 | 10 | 91 | 0.901 | 11 | rs11022775 - rs7396552 | 13373764 - 16988268 | 512 | 3614504 |
| ROH268 | 2 | 26 | 0.606 | 11 | rs10833635 - rs11027556 | 21830364 - 23861558 | 338 | 2031194 |
| ROH269 | 28 | 303 | 0.309 | 11 | rs2716522 - rs831620 | 24869249 - 33709273 | 1172 | 8840024 |
| ROH270 | 49 | 387 | 0.385 | 11 | rs2137344 - rs12281232 | 37103420 - 40155064 | 330 | 3051644 |
| ROH271 | 14 | 105 | 0.528 | 11 | rs4075903 - rs11037740 | 40207923 - 43952762 | 633 | 3744839 |
| ROH272 | 234 | 2026 | 0.464 | 11 | rs11038229 - rs650258 | 44985936 - 60832282 | 1379 | 15846346 |
| ROH273 | 33 | 307 | 0.758 | 11 | rs509360 - rs7931342 | 61548559 - 68994497 | 810 | 7445938 |
| ROH274 | 24 | 229 | 0.719 | 11 | rs11237691 - rs947999 | 70693797 - 76347032 | 770 | 5653235 |
| ROH275 | 7 | 59 | 0.864 | 11 | rs1149618 - rs613011 | 76500875 - 78725925 | 296 | 2225050 |
| ROH276 | 28 | 259 | 0.794 | 11 | rs10792532 - rs10501631 | 80727085 - 86604406 | 1211 | 5877321 |
| ROH277 | 45 | 478 | 0.233 | 11 | rs7122009 - rs689825 | 86710510 - 94666818 | 1174 | 7956308 |
| ROH278 | 7 | 44 | 0.383 | 11 | rs10765800 - rs7934855 | 95962582 - 98733359 | 464 | 2770777 |
| ROH279 | 37 | 381 | 0.376 | 11 | rs518382 - rs643122 | 100931117 - 112687469 | 1856 | 11756352 |
| ROH280 | 3 | 24 | 0.850 | 11 | rs6589426 - rs7131355 | 114256749 - 116077370 | 349 | 1820621 |
| ROH281 | 0 | 15 | 0.197 | 11 | rs4307756 - rs11216517 | 116503644 - 117595511 | 249 | 1091867 |
| ROH282 | 0 | 17 | 0.169 | 11 | rs2155854 - rs12795576 | 118026675 - 119205830 | 154 | 1179155 |
| ROH283 | 0 | 12 | 0.248 | 11 | rs1148115 - rs2846572 | 123494841 - 124497410 | 239 | 1002569 |
| ROH284 | 3 | 14 | 0.300 | 12 | rs7970020 - rs686394 | 9021525 - 10075075 | 204 | 1053550 |
| ROH285 | 5 | 23 | 0.171 | 12 | rs7959064 - rs11054425 | 10284158 - 11864281 | 270 | 1580123 |
| ROH286 | 1 | 19 | 0.452 | 12 | rs12314703 - rs2417550 | 14992226 - 16408222 | 220 | 1415996 |
| ROH287 | 1 | 12 | 0.775 | 12 | rs4764259 - rs11057006 | 16434444 - 16750931 | 84 | 316487 |
| ROH288 | 3 | 99 | 0.016 | 12 | rs11057009 - rs7316716 | 16772801 - 19953193 | 517 | 3180392 |
| ROH289 | 7 | 40 | 0.271 | 12 | rs11045356 - rs2632487 | 20792101 - 22933285 | 502 | 2141184 |
| ROH290 | 7 | 46 | 0.445 | 12 | rs7975710 - rs299441 | 26534066 - 29660204 | 675 | 3126138 |
| ROH291 | 120 | 1118 | 0.515 | 12 | rs33233 - rs7958908 | 31030037 - 47940764 | 2175 | 16910727 |
| ROH292 | 30 | 276 | 0.802 | 12 | rs11168236 - rs1027827 | 48178604 - 51925305 | 486 | 3746701 |
| ROH293 | 2 | 29 | 0.503 | 12 | rs673127 - rs4759062 | 53101668 - 54434908 | 221 | 1333240 |
| ROH294 | 47 | 477 | 0.361 | 12 | rs1874309 - rs168294 | 54950227 - 63116751 | 1088 | 8166524 |
| ROH295 | 3 | 32 | 0.764 | 12 | rs337520 - rs9652019 | 63163019 - 64465309 | 218 | 1302290 |
| ROH296 | 5 | 39 | 0.768 | 12 | rs11609829 - rs7968902 | 64732294 - 66363070 | 200 | 1630776 |
| ROH297 | 3 | 29 | 0.886 | 12 | rs10784757 - rs10506599 | 69508022 - 71022883 | 394 | 1514861 |
| ROH298 | 43 | 395 | 0.767 | 12 | rs7956954 - rs11613246 | 71822686 - 76035715 | 578 | 4213029 |
| ROH299 | 4 | 27 | 0.598 | 12 | rs1368579 - rs300489 | 76428534 - 78485994 | 370 | 2057460 |
| ROH300 | 145 | 1245 | 0.523 | 12 | rs10506773 - rs4503583 | 78583071 - 92308227 | 1701 | 13725156 |
| ROH301 | 3 | 18 | 0.520 | 12 | rs2540514 - rs10777847 | 96491074 - 97693279 | 235 | 1202205 |
| ROH302 | 0 | 11 | 0.269 | 12 | rs1531650 - rs169296 | 98579440 - 99244049 | 132 | 664609 |
| ROH303 | 5 | 52 | 0.738 | 12 | rs11109655 - rs7956620 | 99286617 - 101447641 | 402 | 2161024 |
| ROH304 | 1 | 20 | 0.420 | 12 | rs2241553 - rs1353769 | 102079973 - 103358503 | 200 | 1278530 |
| ROH305 | 4 | 41 | 0.786 | 12 | rs1215597 - rs7310199 | 106532072 - 108572516 | 323 | 2040444 |
| ROH306 | 76 | 766 | 0.254 | 12 | rs10778670 - rs4767066 | 109322744 - 113748702 | 445 | 4425958 |
| ROH307 | 19 | 175 | 0.841 | 12 | rs487818 - rs1263990 | 119929799 - 124845436 | 584 | 4915637 |
| ROH308 | 5 | 31 | 0.445 | 13 | rs5027700 - rs10507431 | 35051477 - 36402079 | 202 | 1350602 |
| ROH309 | 5 | 28 | 0.332 | 13 | rs1980730 - rs1555629 | 37918173 - 40421628 | 507 | 2503455 |
| ROH310 | 10 | 65 | 0.344 | 13 | rs7326616 - rs7995159 | 41009794 - 43434768 | 452 | 2424974 |
| ROH311 | 22 | 197 | 0.869 | 13 | rs1536761 - rs7329171 | 47587422 - 51537048 | 647 | 3949626 |
| ROH312 | 140 | 1218 | 0.607 | 13 | rs1022960 - rs1413137 | 51724935 - 71284358 | 2917 | 19559423 |
| ROH313 | 5 | 13 | 0.013 | 13 | rs9542535 - rs4884998 | 71493149 - 72834892 | 238 | 1341743 |
| ROH314 | 73 | 711 | 0.409 | 13 | rs7335576 - rs9589090 | 76043318 - 91748928 | 2485 | 15705610 |
| ROH315 | 42 | 440 | 0.289 | 13 | rs9590220 - rs9556813 | 95906694 - 98409084 | 409 | 2502390 |
| ROH316 | 4 | 30 | 0.738 | 13 | rs17574743 - rs7997714 | 99290386 - 100555353 | 315 | 1264967 |
| ROH317 | 0 | 12 | 0.248 | 13 | rs2217903 - rs610678 | 102300017 - 102852448 | 146 | 552431 |
| ROH318 | 3 | 24 | 0.850 | 14 | rs202968 - rs1018542 | 27729100 - 29853054 | 275 | 2123954 |
| ROH319 | 20 | 97 | 0.011 | 14 | rs179539 - rs2776612 | 31233659 - 32524419 | 194 | 1290760 |
| ROH320 | 12 | 69 | 0.154 | 14 | rs12050125 - rs927287 | 34838295 - 36924790 | 262 | 2086495 |
| ROH321 | 90 | 900 | 0.235 | 14 | rs712335 - rs7493885 | 37280489 - 51317418 | 2100 | 14036929 |
| ROH322 | 6 | 48 | 0.789 | 14 | rs1253706 - rs210361 | 52400377 - 54164036 | 340 | 1763659 |
| ROH323 | 6 | 38 | 0.430 | 14 | rs3742560 - rs2183628 | 55106083 - 56390908 | 199 | 1284825 |
| ROH324 | 138 | 1048 | 0.044 | 14 | rs17092455 - rs2877796 | 57376044 - 72882983 | 2743 | 15506939 |
| ROH325 | 4 | 85 | 0.079 | 14 | rs7161351 - rs1009392 | 72970502 - 75755192 | 419 | 2784690 |
| ROH326 | 9 | 59 | 0.382 | 14 | rs207801 - rs10144955 | 77676449 - 82359784 | 996 | 4683335 |
| ROH327 | 5 | 40 | 0.807 | 14 | rs1263475 - rs8016533 | 83477238 - 85585139 | 343 | 2107901 |
| ROH328 | 4 | 42 | 0.751 | 14 | rs7151326 - rs3934197 | 86387845 - 88778105 | 373 | 2390260 |
| ROH329 | 0 | 10 | 0.292 | 14 | rs11622455 - rs10135444 | 91669929 - 92152570 | 96 | 482641 |
| ROH330 | 1 | 11 | 0.840 | 14 | rs3783925 - rs7141441 | 93411904 - 94233400 | 126 | 821496 |
| ROH331 | 8 | 89 | 0.544 | 14 | rs12586334 - rs8013773 | 101888862 - 104636584 | 324 | 2747722 |
| ROH332 | 12 | 96 | 0.704 | 15 | rs3101644 - rs7174078 | 27768133 - 29447147 | 198 | 1679014 |
| ROH333 | 7 | 45 | 0.414 | 15 | rs7183354 - rs4779527 | 29928756 - 31736091 | 235 | 1807335 |
| ROH334 | 79 | 735 | 0.619 | 15 | rs2047943 - rs1843089 | 39518016 - 46606631 | 839 | 7088615 |
| ROH335 | 46 | 338 | 0.194 | 15 | rs1484197 - rs1878193 | 46667965 - 53709528 | 1154 | 7041563 |
| ROH336 | 44 | 299 | 0.080 | 15 | rs1398003 - rs12909385 | 54623246 - 57697075 | 491 | 3073829 |
| ROH337 | 15 | 140 | 0.831 | 15 | rs902031 - rs1034505 | 63299362 - 66396554 | 365 | 3097192 |
| ROH338 | 16 | 124 | 0.579 | 15 | rs266366 - rs4468552 | 67221533 - 68765935 | 261 | 1544402 |
| ROH339 | 5 | 28 | 0.332 | 15 | rs4108 - rs437569 | 68951569 - 70098476 | 147 | 1146907 |
| ROH340 | 135 | 1062 | 0.115 | 15 | rs12437565 - rs16970006 | 70478116 - 78970259 | 1105 | 8492143 |
| ROH341 | 47 | 381 | 0.493 | 15 | rs7164765 - rs7175208 | 81691199 - 87285730 | 864 | 5594531 |
| ROH342 | 5 | 67 | 0.376 | 16 | rs11862729 - rs8051552 | 14146098 - 17296549 | 379 | 3150451 |
| ROH343 | 2 | 31 | 0.444 | 16 | rs11074431 - rs2926362 | 20158159 - 22330038 | 225 | 2171879 |
| ROH344 | 6 | 41 | 0.536 | 16 | rs7201219 - rs9941272 | 27919186 - 31957368 | 264 | 4038182 |
| ROH345 | 11 | 66 | 0.216 | 16 | rs12596272 - rs1540620 | 35142792 - 50292343 | 388 | 15149551 |
| ROH346 | 2 | 9 | 0.373 | 16 | rs8056747 - rs421859 | 55906487 - 56575651 | 105 | 669164 |
| ROH347 | 7 | 40 | 0.271 | 16 | rs1482243 - rs16964398 | 59605650 - 62265048 | 367 | 2659398 |
| ROH348 | 4 | 10 | 0.022 | 16 | rs288579 - rs8044825 | 62577626 - 63476324 | 131 | 898698 |
| ROH349 | 3 | 52 | 0.255 | 16 | rs16966585 - rs39564 | 63810244 - 65829616 | 332 | 2019372 |
| ROH350 | 75 | 612 | 0.407 | 16 | rs12918939 - rs11075971 | 66140559 - 73197239 | 675 | 7056680 |
| ROH351 | 2 | 44 | 0.197 | 16 | rs7500512 - rs8048453 | 74108894 - 76564432 | 374 | 2455538 |
| ROH352 | 0 | 11 | 0.269 | 16 | rs12599583 - rs1025318 | 76605753 - 77268935 | 130 | 663182 |
| ROH353 | 2 | 52 | 0.120 | 16 | rs4782486 - rs7196459 | 88646177 - 90141477 | 221 | 1495300 |
| ROH354 | 5 | 21 | 0.121 | 17 | rs1051007 - rs2042005 | 4636813 - 5733558 | 220 | 1096745 |
| ROH355 | 22 | 220 | 0.588 | 17 | rs1981650 - rs1871323 | 15092264 - 21810779 | 668 | 6718515 |
| ROH356 | 26 | 177 | 0.186 | 17 | rs7220080 - rs1076186 | 25589184 - 29921253 | 387 | 4332069 |
| ROH357 | 4 | 18 | 0.207 | 17 | rs714895 - rs17138297 | 33104093 - 34979921 | 221 | 1875828 |
| ROH358 | 61 | 543 | 0.783 | 17 | rs4795223 - rs2119930 | 36122227 - 47514039 | 1290 | 11391812 |
| ROH359 | 19 | 173 | 0.862 | 17 | rs4794293 - rs2958899 | 49741155 - 52869228 | 500 | 3128073 |
| ROH360 | 63 | 497 | 0.316 | 17 | rs8074980 - rs8069926 | 56010960 - 64711817 | 912 | 8700857 |
| ROH361 | 3 | 22 | 0.745 | 17 | rs8072277 - rs2278825 | 72807021 - 74163478 | 157 | 1356457 |
| ROH362 | 3 | 9 | 0.087 | 18 | rs4796957 - rs12969041 | 12267865 - 13191184 | 131 | 923319 |
| ROH363 | 3 | 13 | 0.250 | 18 | rs6505831 - rs786045 | 13606170 - 14949001 | 133 | 1342831 |
| ROH364 | 10 | 92 | 0.887 | 18 | rs1849273 - rs1421117 | 15122502 - 21942543 | 367 | 6820041 |
| ROH365 | 4 | 26 | 0.551 | 18 | rs9951749 - rs7227201 | 22757449 - 24141376 | 226 | 1383927 |
| ROH366 | 67 | 537 | 0.360 | 18 | rs1420950 - rs7238127 | 25127853 - 45577937 | 3567 | 20450084 |
| ROH367 | 3 | 25 | 0.898 | 18 | rs9960784 - rs9948424 | 46419946 - 48233078 | 420 | 1813132 |
| ROH368 | 36 | 283 | 0.445 | 18 | rs1435999 - rs585491 | 49012000 - 55167028 | 1021 | 6155028 |
| ROH369 | 1 | 22 | 0.363 | 18 | rs3862706 - rs8088963 | 57728033 - 59047314 | 209 | 1319281 |
| ROH370 | 5 | 18 | 0.064 | 18 | rs4941055 - rs11152368 | 59422272 - 60763112 | 275 | 1340840 |
| ROH371 | 1 | 11 | 0.840 | 18 | rs2048028 - rs1421548 | 61511572 - 62055124 | 99 | 543552 |
| ROH372 | 6 | 49 | 0.823 | 18 | rs470497 - rs9967050 | 63031505 - 64825748 | 252 | 1794243 |
| ROH373 | 2 | 8 | 0.298 | 18 | rs4316858 - rs8083908 | 66570423 - 67144161 | 97 | 573738 |
| ROH374 | 2 | 9 | 0.373 | 18 | rs2141834 - rs7237766 | 68178918 - 69046122 | 148 | 867204 |
| ROH375 | 12 | 43 | 0.004 | 19 | rs10415904 - rs1133330 | 9314135 - 12772090 | 412 | 3457955 |
| ROH376 | 50 | 343 | 0.070 | 19 | rs4808801 - rs7250772 | 18571141 - 30024773 | 870 | 11453632 |
| ROH377 | 7 | 23 | 0.016 | 19 | rs12971522 - rs8109247 | 31306594 - 33490685 | 309 | 2184091 |
| ROH378 | 28 | 442 | 0.002 | 19 | rs10410294 - rs7259197 | 36097328 - 39542089 | 355 | 3444761 |
| ROH379 | 6 | 48 | 0.789 | 19 | rs338594 - rs10402946 | 41703660 - 44139182 | 202 | 2435522 |
| ROH380 | 2 | 14 | 0.745 | 19 | rs344772 - rs7255066 | 44179956 - 45146103 | 168 | 966147 |
| ROH381 | 0 | 11 | 0.269 | 20 | rs6078273 - rs1998032 | 11623168 - 12255587 | 120 | 632419 |
| ROH382 | 7 | 56 | 0.772 | 20 | rs17812299 - rs6043125 | 13283622 - 15161670 | 383 | 1878048 |
| ROH383 | 0 | 12 | 0.248 | 20 | rs6080967 - rs6081411 | 18067362 - 18905455 | 145 | 838093 |
| ROH384 | 53 | 393 | 0.181 | 20 | rs1122098 - rs2404447 | 20378708 - 22783872 | 292 | 2405164 |
| ROH385 | 75 | 868 | 0.017 | 20 | rs3004119 - rs198488 | 23489678 - 36276257 | 985 | 12786579 |
| ROH386 | 8 | 86 | 0.607 | 20 | rs2066252 - rs1980594 | 36937246 - 38870253 | 311 | 1933007 |
| ROH387 | 2 | 45 | 0.185 | 20 | rs742748 - rs6016677 | 39293397 - 40700626 | 239 | 1407229 |
| ROH388 | 1 | 19 | 0.452 | 20 | rs6031782 - rs6073995 | 43383832 - 44668008 | 195 | 1284176 |
| ROH389 | 1 | 26 | 0.273 | 20 | rs16993748 - rs1555317 | 47037310 - 48643650 | 284 | 1606340 |
| ROH390 | 2 | 14 | 0.745 | 21 | rs2827312 - rs2827986 | 23631676 - 24616086 | 152 | 984410 |
| ROH391 | 5 | 45 | 0.936 | 21 | rs2829272 - rs961302 | 26070229 - 27782968 | 289 | 1712739 |
| ROH392 | 41 | 348 | 0.700 | 21 | rs1864657 - rs7281210 | 29157872 - 32485200 | 518 | 3327328 |
| ROH393 | 1 | 16 | 0.567 | 21 | rs11701165 - rs2834070 | 33274026 - 34387452 | 226 | 1113426 |
| ROH394 | 1 | 11 | 0.840 | 21 | rs2834101 - rs2032314 | 34515010 - 35354523 | 160 | 839513 |
| ROH395 | 4 | 17 | 0.173 | 22 | rs2541943 - rs5756808 | 21376092 - 22437729 | 132 | 1061637 |
| ROH396 | 1 | 13 | 0.716 | 22 | rs1003784 - rs2295102 | 24220358 - 25209753 | 109 | 989395 |
| ROH397 | 128 | 999 | 0.106 | 22 | rs6005585 - rs9609560 | 28109665 - 32856999 | 743 | 4747334 |
| ROH398 | 0 | 12 | 0.248 | 22 | rs9609893 - rs5755469 | 34359717 - 35369739 | 201 | 1010022 |

**Supplementary Table 1**: Details of location, length and frequency of all 398 ROH found
